# Low density DNA microarray for detection of most frequent *TP53 *missense point mutations

**DOI:** 10.1186/1472-6750-5-8

**Published:** 2005-02-15

**Authors:** Angélica Rangel-López, Rogelio Maldonado-Rodríguez, Mauricio Salcedo-Vargas, Juana Mercedes Espinosa-Lara, Alfonso Méndez-Tenorio, Kenneth L Beattie

**Affiliations:** 1 Escuela Nacional de Ciencias Biológicas IPN, México, 11340, D. F., México, 11340, D. F., México; 2 Unidad de Investigación Médica en Enfermedades Oncológicas. Hospital de Oncología. Centro Médico Nacional S XXI. IMSS, México, D. F., México; 3 Amerigenics, Inc., 1326 Open Range Rd., Crossville, TN 38555 USA

## Abstract

**Background:**

We have developed an oligonucleotide microarray (genosensor) utilizing a double tandem hybridization technique to search for 9 point mutations located in the most frequently altered codons of the *TP53 *gene. Isolated and multiplexed PCR products, 108 and 92 bp long, from exons 7 and 8, respectively, were obtained from 24 different samples. Single-stranded target DNA was then prepared from isolated or multiplexed PCR products, through cyclic DNA synthesis. Independent ssDNA's were annealed with the corresponding pairs of labeled stacking oligonucleotides to create partially duplex DNA having a 7-nt gap, which contains the sequence that will be interrogated by the capture probes forming double tandem hybridization. In the case of multiplexed ssPCR products, only two stacking oligonucleotides were added per target, therefore the gap for the PCR products having two consecutive codons to be interrogated in exon 7 was 12 nt long, so only single tandem hybridization was produced with these respective probes.

**Results:**

18 codon substitutions were found by DNA sequencing. In 13 of them a perfect correlation with the pattern of hybridization was seen (In 5 no signal was seen with the wt probe while a new signal was seen with the appropriate mutant probe, and in 8 more, as expected, no signal was seen with any probe due to the absence of the corresponding probe in the array). In 3 other cases a mutation was falsely suggested by the combination of the absence of the wild type signal along with a false signal in the other probe. In the other 2 cases the presence of the mutation was not detected due to the production of a false hybridization signal with the wild type probe. In both cases (false mutation or no mutation detected) relatively stable mismatched target/probe duplexes should be formed. These problems could be avoided by the addition of probes to improve the performance of the array.

**Conclusion:**

Our results demonstrate that a simple *TP53 *microarray employing short (7-mer) probes, used in combination with single or double tandem hybridization approach and a simple or multiplex target preparation method, can identify common *TP53 *missense mutations from a variety of DNA sources with good specificity.

## Background

Cancer is a group of diseases characterized by uncontrolled cell growth. According to The World Cancer Report, cancer rates could further increase by 50% to 15 million new cases in the year 2020 [1]. The chances of surviving the onset of some common cancers depend largely on how early they are detected in addition to how well they are treated.

The progression of mammalian cells towards malignancy is an evolutionary process that involves an accumulation of mutations at the molecular and chromosomal level. A candidate for involvement in this process is the tumor suppressor gene *TP53 *(MIM # 191170) which encodes a transcription factor (p53 protein) with cancer inhibiting properties. This gene is the most frequently mutated gene in human cancer. Between 30% and 70% of cancers of almost every organ and histological subtype have a point mutation in one of the two *TP53 *gene copies together with loss of the other copy [2-4]. In many cancers the distribution of mutations along the *TP53 *gene is tumor-specific, clustered between exons 5, 7 and 8. This region is highly conserved throughout evolution [3]. In this domain, six mutational "hotspots" have been identified at codons 175, 245, 248, 249, 273, and 282. The mutational profile of the *TP53 *gene is different between cancers, for example, codons 157, 158, 248, 249, and 273 have been designated as *TP53 *mutational hotspots in lung cancer [5,6], while in breast cancer the mutational hotspots are in codons, 175, 245, 248, 249 and 273 [7]. On the contrary, in cervical cancer the frequency of the p53 mutations is very low. [8].

Because mutations in the *TP53 *gene are so common in human tumors and have been widely reported in the literature, extensive mutational databases exist, such as that maintained by IARC [9]. Walker et al. [10], have utilized the IARC TP53 mutation database to define 73 "hotspots" for mutation in *TP53 *related to changes in protein structure and function. Martin et al. [11] performed a systematic automated analysis of the effects of TP53 mutations on the structure of the core domain of the p53 protein to functionally classify the various types of p53 mutants according to predicted effects on protein folding or DNA-protein interactions. Most mutations are currently identified by conventional methods of polymorphism detection and DNA sequencing [12]. A growing number of nucleic acid hybridization techniques have recently been applied to biomedical problems, including development of DNA probe arrays for detection of *TP53 *mutations [13-15]. In many applications, DNA chips containing surface-bound oligonucleotides (probes) are used to interrogate sample (target) sequence information through complementary recognition (hybridization) in a highly parallel fashion [16]. Major applications of DNA microarrays include gene expression profiling [17,18] and gene mutation analysis [19,20]. These techniques allow parallel analysis of multiple DNA samples. Mutation studies with DNA microarrays are still at an early stage and are in continuous development [21,22]. Although the field of microarray assays is still in a relatively early stage, it is generally anticipated that microarray assays will offer decreased cost and faster results, compared with the traditional, more labor intensive dideoxy sequencing approach. We recently reported a novel tandem hybridization strategy and its application for identification of mutations. Reliable discrimination of point mutations has been achieved by double tandem hybridization with a set of seven-mer probes [23,24]. In the present work we designed a novel small format DNA chip (genosensor array) to search for nine point mutations in codons 248, 249 and 273 of the *TP53 *gene.

## Results

### PCR products and reference hybridization patterns

PCR products from exon 7 (codons 248 and 249) and exon 8 (codon 273) were obtained from each type of DNA sources listed in Materials and Methods. Figure 2A shows gel electrophoretic analysis of the fragments of 108 and 92 bp corresponding to exons 7 and 8, respectively. To prepare ssDNA targets PCR products were used as templates for cycle synthesis of a target strand, using the reverse PCR primer in single or multiplexed reactions cycled 25 times. The production of single-stranded target DNA's was assessed by its change in electrophoretic mobility in 4% agarose gels (Figure 2B).

The reference hybridization patterns produced using synthetic DNA targets are shown in Figures. 3A and 3B. As expected, all the synthetic targets, including those of the negative controls, yielded hybridization signals with their respective ("perfect-match") probes. Pairs of wild-type with each mutant synthetic target sequences (1:1 mixtures) were also hybridized to reproduce heterozygous conditions. All gave the expected signals, (Figure 3B).

### Hybridization of DNA targets

Hybridization of individual or multiplexed ssDNA targets prepared from the 24 DNA samples (that correspond to 72 codons in 24 samples) was performed with similar results. Base substitutions were seen in 11 out of the 24 samples tested (Table 3). One, two or three codons were altered in 5 (P6, L1, L2, B3 and B6), 5 (P1, L4, L7, L9 and L10) and 1 (L8) samples, respectively. In the cases of the samples having two or three codons affected, at least one of the base substitutions was silent. In the other 13 samples only WT sequences were identified (data not shown). Representative hybridization patterns are shown in Figure. 4A and the overall results are summarized in Table 3. The DNA samples hybridized with one or two probes from each codon, corresponding to the homozygous or heterozygous condition, respectively. Among the 24 different samples multiplexed or individually tested in the microarray, six single base susbstitutions in codon 248 were found by sequencing the PCR product. All of them corresponded to base substitutions keeping the original aminoacid (Arginine). The same number of point mutations were also seen in codon 249 by DNA sequencing. However, no probes to search for these base substitutions were placed in the DNA microarray. Six point mutations in codon 273 were obtained. All of them, except the CGT→CCT change observed in plasmid P6, were searched with the set of probes contained in the DNA microarray. In this codon all the base substitutions are producers of aminoacid substitution.

## Discussion

The *TP53 *tumor suppressor gene has proven to be one of the cellular genes most often mutated in human neoplasias. Analysis of the mutational events that target the *TP53 *gene has revealed evidence for both exogenous and endogenous mutational mechanisms. This gene mutational spectrum suggests evidence for a direct causal effect of tobacco smoke in lung cancer [26,27], aflatoxin B1 in liver cancer [28]. Therefore, identification of mutations in the *TP53 *gene may play an important role in the diagnosis, staging, and management of the cancer patients.

More than 70% of the molecular studies have focused on the central region of the *TP53 *gene, more specifically on exons 5 through 8 which encode the DNA binding domain [3]. Thus, missense mutations in this region can cause a modification that may alter DNA binding. It is therefore essential that clinical trials aim to accurately establish the effect of *TP53 *mutations on all clinical parameters. One approach is the p53 GeneChip of Affymetrix used to identify mutations in this gene [13,14]. However, this technology is expensive and poorly available to many clinicians, which makes it necessary to develop new mutation detection methods [29,30].

The updated IARC TP53 mutation database [38] shows that more than 1700 different point mutations, which are associated to cancers, are distributed along the *TP53 *gene. *TP53 *somatic mutations are found in most types of sporadic human cancers at various frequencies (from 20% to 60%) [13,14]. The database also shows that approximately 20% of all mutations are located in the five codons (hotspots) in the core domain: 175, 245, 248, 249 and 273. Therefore a primary screen of these codons would be attractive.

Most SNP diagnostic arrays use probes varying from 15 to 20 nt in length with similar Tm values [31]. However, under this circumstance some of the mutant sequences are difficult to discriminate, due to the relatively small destabilizing effect of certain single mismatches in the probe/target duplex [32].

The tandem hybridization method described here offers several advantages over the traditional oligonucleotide array configuration in which each interrogated target sequence is represented by a single surface-tethered probe. Because the long stacking probe targets the analysis to a single unique site within a nucleic acid, direct hybridization analysis of nucleic acid samples of high genetic complexity using short capture probes may be enabled [24].

Recently, we successfully used a double tandem hybridization strategy in the genesensor chip system to discriminate point mutations in codon 634 of RET oncogene in individuals affected by medullary thyroid carcinoma [23]. This DNA chip format involves a target sequence which is annealed with a pair of labeled oligonucleotides (stabilizing oligonucleotides) prior to hybridization to the array of capture probes, forming a single-stranded gap between the stabilizing oligonucleotides and corresponding to the length of the capture probes. The partially duplex product contains the sequences interrogated by the capture probes, and when the sequences between the target DNA and the capture probe (attached to slide) are perfectly complementary, the stability of the hybridization is enhanced due to coaxial stacking of bases between the contiguous ends of the probe and the stabilizing oligonucleotides. The energy involved varies with the identity of the base pairs and the coaxial stacking [22]. Tandem hybridization uses shorter capture probes than traditional oligonucleotide array hybridization, which may contribute to increased discrimination of point mutations because the single base mismatch acts on a less stable duplex formed between the target DNA and the shorter probe. Two additional attractive features inherent to tandem hybridization are that, i) the probability that formation of secondary structure within the single stranded DNA [25] will block accessibility of the target sequence is reduced, and ii) the label needed to reveal the hybridization can be introduced into the two stabilizing oligonucleotides, avoiding the extra step of labeling individual target strands [33,34].

The goal of the present work was to develop a simple format array able to perform a primary screening of the mutations occurring in the most frequently affected codons of the *TP53 *gene.

It has been recently reported that detection of mutations yields variable results depending on the type of DNA sample [35,36]. Is clear that, the sensitivity to detect mutations in TP53, may also differ depending on the method used. In the current study the double tandem hybridization assay was applied to genomic DNA samples extracted from a variety of sources, including blood cells, cell lines and plasmids. There were not any differences in the ability of our system to detect mutations with respect to the different DNA sources.

In the experiments employing synthetic DNA targets hybridized with arrays of mutant and WT probes the expected hybridization signals were seen both in homozygous and heterozygous conditions (Figure. 3). This result indicates that the probes are working properly.

The hybridization of natural targets was done both with isolated and multiplexed PCR products with similar results. Multiplexing was applied in all (PCR reaction ssDNA target preparation, annealing of target with stacking oligonucleotides and hybridization) the steps. So these results are suggesting that multiplexing can be confidently employed for this analysis.

Base changes were found in 11 DNA samples. Two were from plasmid, seven from cell lines and two from blood samples. In 5 of these samples a single codon was affected, in other 5 samples two codons were altered, and in the other sample three codons were affected. Therefore a total of 18 codon alterations were detected. In five of the 18 codon alterations (Samples L2 codon 273, L7 codon 273, L8 codon 273, B3 codon 273 and B6 codon 273) both the absence of hybridization signal with the wild type probe and the production of signal with the appropriated probe was seen. In eight cases (Samples P1 codon 248, P6 codon 273, L8 codons 248 and 249, L9 L8 codons 248 and 249 and L8 codons 248 and 249 and, L10 codons 248 and 249) no hybridization signal was seen even with the wild type sequence. This was due to the absence of the corresponding mutant probe in the array. In three cases (P1 codon 249, L4 codon 249 and L7 codon 249) having either homozygous (sample P1) or heterozygous (samples L4 and L7) genotypes (wtAGG→mutAGA) no hybridization signal with the wild type probe and, an additional false hybridization signal with the probe corresponding to the substitution of guanine for thymine in codon 249 (p-249 G_2_→T) was seen, which could be relatively stable under the conditions tested. Therefore an additional probe, searching by the AGA sequence, should be added in the future array to discriminate between the two alternative silent (AGA) and missense (AGT) genotypes. In the other two codons affected (L1 codon 248 and L4 codon 248) the presence of signal with the wild type probe was seen even when the sequencing was showing a base change (wtCGG→mutCGT in sample L1 or 248 wtCGG→mutCGK in sample L4). In both cases a G:N mismatch is formed which, according to SantaLucia [37], is relatively stable. So, as in the previous two cases the incorporation of additional probes in the array should help to improve performance of the DNA microarray to confidently identify the genotypes.

The base substitution observed in B3 and B6 blood samples is a mutation that substitutes Arginine by Leucine. The presence of this mutation in blood suggests that it could be from germinal origin.

In all the mutations (silent and missense) observed an Arginine was involved, which can be expected because it is coded by 6 codons and it frequently produces non conservative aminoacid substitutions.

The array is able to confidently detect most of the mutations searched, however, additional probes should be added to improve its performance.

## Conclusions

Our results demonstrate that a simple *TP53 *microarray using short (7-mer) probes, used in combination with a double tandem hybridization approach and a simple multiplex fragment preparation method, can identify common *TP53 *point mutations from different DNA sources. All the previous data confirms that the tandem hybridization technology, with the appropriated set of probes, is highly specific and can be confidently employed for diagnostic purposes. This system could provide rapid, economical and accurate mutational screening in the *TP53 *gene. We are presently improving our system by adding probes for these and other *TP53 *mutational hot spots.

## Methods

### IARC TP53 gene database search

The distribution of nucleotidic changes in the *TP53 *gene associated with all cancers war retrieved from the IARC *TP53 *database [38]. Then, the single base substitutions occurring in the 3 exons of *TP53 *most frequently altered in cancers were obtained. Both wild type (accession # U94788) and mutated *TP53 *sequences were used to design the probes, stacking oligonucleotides, PCR primers and synthetic targets.

### Oligonucleotides

**Figure 1:**
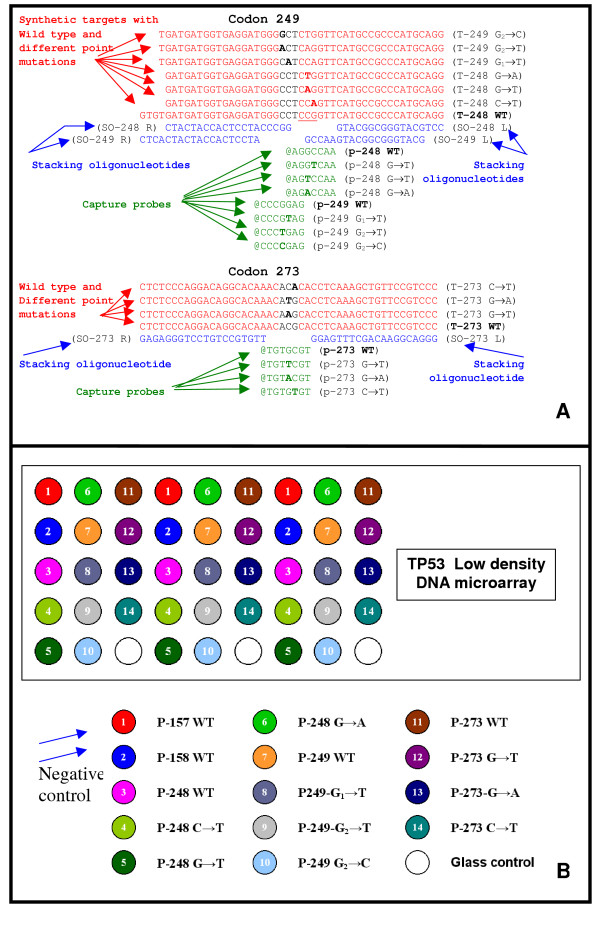
**Layout of the *TP53 *low density DNA microarray**. (A): Names and alignment of stacking oligonucleotides and probes to their respective synthetic wild type target sequence. Bold letters correspond to the nucleotide change in DNA sequence due to the point mutations. (B): Layout of the probe array on the glass slide. The probes were applied to the slide in triplicate as depicted at the top. The numbers correspond to the probes in Table 1.

**Figure 2:**
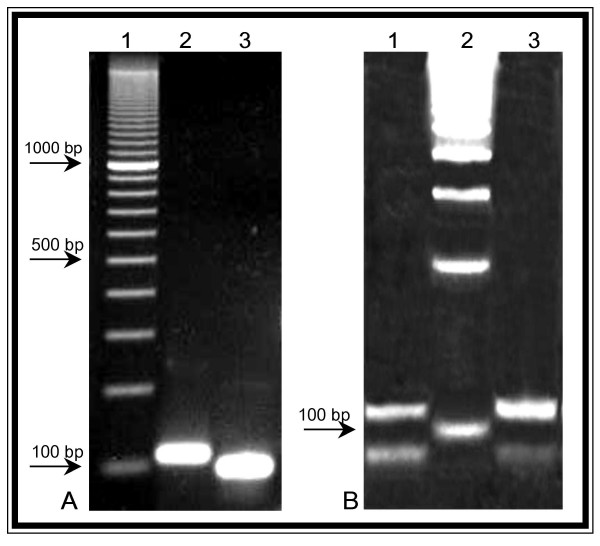
**Electrophoretic analysis of PCR products and single-stranded target DNA produced by "cycle sequencing." **(A): Electrophoresis in 2% agarose gel from PCR reactions of regions from exons 7 and 8 of the *TP53 *gene: 100 bp ladder (Lane 1), 108 bp PCR product from exon 7 (lane 2), 92 bp PCR product from exon 8 (lane 3). (B): Electrophoresis in 4% agarose gel of two multiplex PCR reactions from exons 7 and 8 of the TP53 gene. Lanes 1 and 3 represent the products of two different samples; lane 2 is the 100 bp ladder.

**Figure 3:**
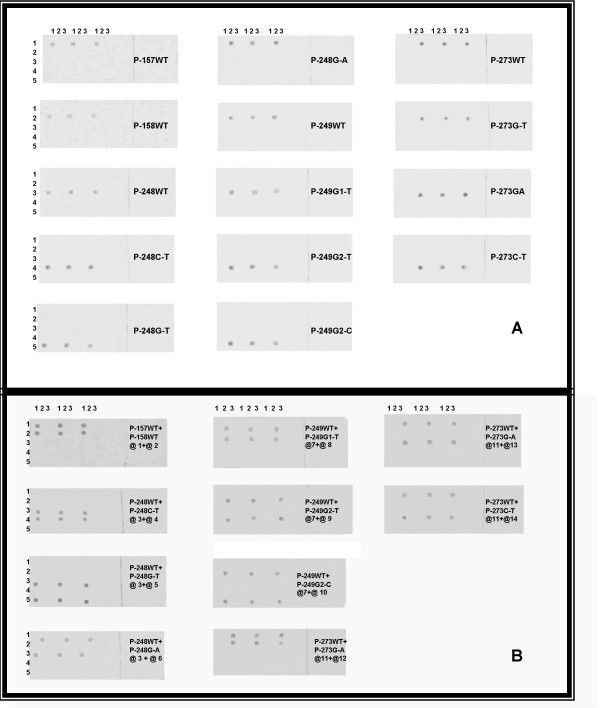
**Hybridization of synthetic targets**. (A) Homozygous pattern. Hybridization pattern produced by synthetic target (ST) on probes (arrayed onto 14 slides). Synthetic targets were annealed with prelabeled stacking oligonucleotides and hybridized with the array of probes having sequence variations. (B) Heterozygous pattern. Hybridization patterns produced by heterozygous combination of synthetic targets (ST) on probes. Equivalent amounts of wild type and mutant synthetic targets were annealed with prelabeled 5'and 3'stacking oligonucleotides, then hybridized with the array of probes having sequence variations (see Table 1).

**Figure 4:**
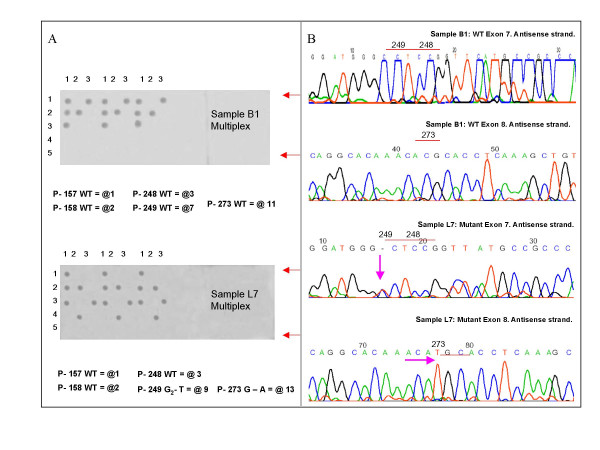
**Hybridization of samples proposed in the multiplex format**. (A). Sample with wild type genotype. Representative experiment of multiplex hybridization assay with sample P1. The red arrows indicates the wild type hybridization signals. The DNA obtained was individually annealed with the two pairs of stacking oligonucleotides and hybridized in one assay. Above Multiplex Assay with sample L7. The hybridized with their respective array of probes having sequence variations. The DNA obtained was annealed the two pairs of stacking oligonucleotides and hybridized in the multiplex format with. (B) The electropherogram showed the sequence wild type of the interest codon. The pink row pointed the signal of the probe in the microarray (point mutation).

Four new PCR primers of 20–25 nt length (Table 1) were used to amplify two DNA fragments of 108 bp (exon 7: codons 248 and 249) and 92 bp (exon 8: codon 273) within the *TP53 *gene. Six stacking oligonucleotides of 17–21 nt length were annealed with their corresponding target DNA's, in three pairs, to produce partially duplex DNA's having a 7-nt gap which is the sequence interrogated by the capture probes (Figure 1A). Fourteen capture probes (seven-mer length; Table 1) were designed, two of them to search the wild type sequences for codons 157 and 158, which were used as negative controls, three more for the wild type 248, 249 and 273 codon sequences and nine to look for mutant sequences. According to our previous experience, 7-mer oligonucleotides with base changes located at central positions were selected as probes in order to yield good discrimination of point mutations by tandem hybridization at room temperature. The probes, which had a 3'-aminolink (3'-aminopropanol) modification for covalent binding to the glass slide surface [24], were purchased from Integrated DNA Technologies Inc. (Coralville, IA, USA).

### Biological samples (DNA targets)

Twenty-four DNA samples from different sources were probed, some having known wild type or mutant *TP53 *sequences and others with unknown *TP53 *genotype. These included eight blood samples (B), two obtained from healthy subjects and six obtained from lung cancer affected patients, and ten established cancer cell lines or recombinant DNA's acquired from the ATCC (L). Two of the cell lines were a generous gift from Dr Mary A Salazar (IIB, UNAM) and one was a gift from Dr José Sullivan (INER, SS, Mexico). The other eight cell lines were provided by the DNA bank from Genomic Oncology Laboratory (CMN S XXI). Finally, six recombinant plasmids (P) harboring *TP53 *(wild type or different point mutations), provided by MSc. Irene Correa (CMN S XXI) were employed to standardize the hybridization arrays. The names, identification numbers, source and known genotypes in the 24 samples studied are described in Table 2

### Target preparation and PCR assay

Genomic DNA was isolated and purified using the Genomic DNA Extraction Kit (Life Technologies Inc., Gaithersburg, MD). DNA concentration was determined by absorbance at 260 nm in a spectrophotometer MBA 2000 (Perkin-Elmer) and by the PicoGreen^® ^dsDNA Quantitation Kit (Molecular Probes).

Plasmid DNA was prepared from bacterial cultures using the alkaline lysis/SDS minipreparation according to the manufacturer's instructions (Life Technologies).

The quality of the DNA preparations was assessed by gel electrophoresis in 1% agarose. All the reagents for PCR reactions were purchased from Promega (Madison WI. USA). The PCR primers are described in Table 1. Oligonucleotides sequences and fragment sizes for each exon were as follows: PCREX7-F and PCREX7-R for a 108 bp product in exon 7, and PCREX8-F and PCREX8-R for a 92 bp fragment in exon 8. The final reaction mixture contained 0.2 mM each dNTP, 50 mM KCl, 10 mM Tris-HCl (pH 8.4), 1.5 mM MgCl_2_, 0.5 μM each primer, 2.5 U of Taq DNA pol (Promega) and purified DNA (50–100 ng) in a final volume of 100 μL. The PCR profile consisted of an initial heating at 94°C for 5 min, followed by 30 cycles of 94°C for 30 s, 55°C for 30 s and 72°C for 30 s, with a final extension step at 72°C for 7 min. The PCR product was assessed by electrophoresis in 1 % agarose gel stained with ethidium bromide.

Single-stranded target DNA was prepared by cycle synthesis as follows. A 30-μL aliquot of PCR product was processed using Ultrafree (Millipore, Bedford, MA) spin filters (30,000 M_r _cutoff), and resuspended in the same volume of HPLC-grade water (OMNI SOLV^®^, EM Science). A 5-μL aliquot of this solution was added to a 100-μL PCR reaction, along with the primer corresponding to the target strand, then incubated for 27 temperature cycles using the same temperature profiles described above. The formation of each amplicon was assessed by mobility shift in agarose gel electrophoresis.

Each one of the six stacking oligonucleotides was 5'-labeled by reaction with T4 polynucleotide kinase (Invitrogen, San Diego, CA) as follows. 5 pmol of each dephosphorylated stacking oligonucleotide; 5X Forward Reaction Buffer (5 μl); T4 Polynucleotide Kinase (10 unit); [γ^32^P]ATP (10 μCi/μl, 3000 Ci/mmol) 2.5 μl (NEN, Boston MA), specific activity 7000 Ci/mmol, diluted in sterile water to 7 μCi/μl, and autoclaved water HPLC-grade to 25 μl. The mix was incubated 10 min at 37°C and the reaction was stopped by adding 5 mM EDTA (2.5 μl).

To prepare labeled partially duplex (gapped) target DNA's, each pair of γ^32^P-labeled stacking oligonucleotides was preannealed with the respective synthetic DNA target, single-stranded PCR fragments or multiplexed, single-stranded PCR products to form a 7-nt gap in each target. The annealing mixture contained 50 μL 20X SSC, 10 μL 1 M Tris-HCl (pH 8.0), 3 μL 0.5 M EDTA, 0.2 pmol of each labeled stacking oligonucleotide, 10 μL of target DNA, and HPLC-grade H_2_O to a final volume of 90 μL. The mixture was incubated at 95°C for 5 min, 45°C for 5 min, then 6°C for 5 min. Excess [γ^32^P]ATP was removed by filtration through an Ultrafree spin filter (3,000 M_r _cutoff) (Millipore. Bedford, MA), and the retained DNA was dissolved in 20 μL of 1X SSC.

In the case of multiplexed targets, only 4 stacking oligonucleotides (SO-248-R, SO-249-L, SO-273R and SO-273-L) were added, one pair for each exon in the study, forming gap sizes of 12 and 7 nt for exons 7 and 8, respectively. Thus, with exon 7 a single tandem hybridization was conducted with the glass-tethered 7-mer capture probes, whereas with exon 8 double tandem hybridization was conducted.

### DNA probe arrays

Glass microscope slides were prepared by soaking in hexane 20 min, then 20 min in absolute ethanol followed by drying 5 h at 90°C. Oligonucleotide probes containing 3'-terminal aminopropanol modification were dissolved in sterile water to a final concentration of 20 μM, and 200 μl droplets of each probe were applied to the glass slides using a model 417 Affymetrix (Santa Clara, CA) arrayer to place submicroliter droplets onto glass slides. Each array contained 14 probes: three wild type sequences and nine mutant sequences corresponding to the *TP53 *targets under study, two negative controls consisting of 7-mers targeted to the wild type sequences from codons 157 and 158 of *TP53 *gene, plus one blank spot without probe. As depicted in Fig. 1B, each *TP53 *capture probe was arrayed in triplicate (to assess the reproducibility of the results) in an upward direction starting from position 1 and the printing was divided into three groups. Positions 1–14 were printed first, then the pattern was repeated twice more across the slide. Position 15 was left empty (nothing at all was applied) and served as a negative glass control. After spotting, each slide was washed with water to remove unbound oligonucleotides, air-dried, then stored in a dessicator at room temperature. This attachment procedure results in a surface density of 10^10^-10^11 ^molecules mm^2^, which corresponds to intermolecular spacing of about 30–100 Å across the surface [25].

### Hybridization experiments

Just prior to hybridization the slides were soaked for 1 h at room temperature with blocking agent (10 mM tripolyphosphate) and then rinsed twice with water and air-dried. The hybridization cocktail contained: 118 μL 5 M tetramethyl-ammonium chloride (TMAC) (Life Technologies), 9 μL 1 M Tris-HCl (pH 8.0) (Life Technologies), 0.72 μL 0.5 M EDTA, 1.8 μL 10% (w/v) sodium dodecyl sulfate, 14.4 μL 40% (w/v) polyethylene glycol, 20 μL labeled target DNA in 1X SSC, and 35.3 μL HPLC-grade water. A 40-μL aliquot of this cocktail was placed onto each array and a cover slip was applied. Slides were incubated in a humid chamber for 3 h at 25°C. After hybridization, the slides were washed by dipping several times in the same hybridization solution without polyethylene glycol and DNA, air-dried, and then wrapped in plastic film and placed against X-ray film (Kodak BioMax) for autoradiography. Detection and quantitation of ^32^P-labeled target molecules bound across the hybridization array was performed with a scanner (HP Scanjet 4400 c), and analysis of densitometric images was performed using ScanAnalyze 2 software (Stanford University).

To provide confidence in the interpretation of the signals produced by human samples, reference hybridization patterns were prepared using a full set of synthetic targets. For this purpose, thirteen synthetic targets (44–50 nt), representing the wild type and mutant sequences, were used independently and in combination, to represent homozygous and heterozygous conditions.

### Sequencing of PCR products

The PCR products obtained were purified using the QIAEX II kit (QIAGEN Inc. USA). For each PCR product the presence or absence of a mutation in exons 7 and 8 was demonstrated via sequencing using the Big Dye Terminator kit (Perkin-Elmer) and a model 373 automated DNA sequencer (Instruments Core Center of Health Research Council- IMSS).

## Authors' contributions

ARL performed all molecular assays, data collection and analysis, and drafted the manuscript. RMR conceived and designed the study, coordinated and managed the study, performed data analysis and participated in drafting the manuscript. MSV conceived and designed the study, coordinated and managed the study, performed data analysis and participated in drafting the manuscript. JMEL participated in the design and coordination of the study. AMT designed the oligonucleotide probes, performed bioinformatic analysis of targets, and participated in drafting the manuscript. KLB assisted in coordination of the study, data analysis, and drafting the manuscript. All authors read and approved the final manuscript.

## Figures and Tables

**Table 1:** Primers, stacking oligonucleotides, synthetic targets and capture probes used.

**Primers**	
Name	Sequence (5'→3')
PCREX7-F	GCTCTGACTGTACCACCATCCA
PCREX7-R	ACCTGGAGTCTTCCAGTGTGA
PCREX8-F	ACTGGGACGGAACAGCTTTGA
PCREX8-R	GCTCCCCTTTCTTGCGGAGAT
	
**Stacking oligonucleotides**	
Name	Sequence (5'→3')
LSO108–248	CCTGCATGGGCGGCATG
RSO108–248	GGCCCATCCTCACCATCATC
LSO108–249	GCATGGGCGGCATGAACCG
RSO108–249	ATCCTCACCATCATCACTC
LSO92–273	GGGACGGAACAGCTTTGAGG
RSO92–273	TTGTGCCTGTCCTGGGAGA
	
**Synthetic targets**	
Name	Sequence (5'→3')
ST-157,158WT	GGTGTGGGGGCGGGCCGTGGGCGCAGGCGCGGTACCGGTAGATGTTCGTC
ST-248,249WT	GTGTGATGATGGTGAGGATGGGCCTCCGGTTCATGCCGCCCATGCAGG
ST-248C→T	GATGATGGTGAGGATGGGCCTCCAGGTCATGCCGCCCATGCAGG
ST-248G_1_→T	GATGATGGTGAGGATGGGCCTCAGGTTCATGCCGCCCATGCAGG
ST-248G_1_→A	GATGATGGTGAGGATGGGCCTCTGGTTCATGCCGCCCATGCAGG
ST-249G_1_→T	TGATGATGGTGAGGATGGGCATCCAGTTCATGCCGCCCATGCAGG
ST-249G_2_→T	TGATGATGGTGAGGATGGGACTCAGGTTCATGCCGCCCATGCAGG
ST-249G_2_→C	TGATGATGGTGAGGATGGGGCTCTGGTTCATGCCGCCCATGCAGG
ST-273WT	CTCTCCCAGGACAGGCACAAACACGCACCTCAAAGCTGTTCCGTCCC
ST-273G→T	CTCTCCCAGGACAGGCACAAACAAGCACCTCAAAGCTGTTCCGTCCC
ST-273G→A	CTCTCCCAGGACAGGCACAAACATGCACCTCAAAGCTGTTCCGTCCC
ST-273C→T	CTCTCCCAGGACAGGCACAAACACACACCTCAAAGCTGTTCCGTCCC
	
**Capture probes**	
Name	Sequence (5'→3')
p-157WT	CGCGTCC@
p-158WT	GTCCGCG@
p-248WT	AACCGGA@
p-248C→T	AACTGGA@
p-248G_1_→T	AACCTGA@
p-248G_1_→A	AACCAGA@
p-249WT	GAGGCCC@
P-249G_1_→T	GATGCCC@
P-249G_2_→T	GAGTCCC@
P-249G_2_→C	GAGCCCC@
P-273WT =	TGCGTGT@
P-273-G→T	TGCTTGT@
P-273G→A	TGCATGT@
P-273C→T	TGTGTGT@

@ = 3'-aminopropanol modification for glass attachment.

Base changes are represented in bold

**Table 2:** Biological samples tested.

**ID**	**Name**	**Source**	**The genotype was previously assumed as:**
P1	pp53	Plasmid	Unknown
P2	pSCX3	Plasmid	Unknown
P3	pM47	Plasmid	Unknown
P4	pC53	Plasmid	Unknown
P5	XbaI	Plasmid	Unknown
P6	C1	Plasmid	Unknown
L1	A-427	Human cell line	Unknown
L2	C33 A	Human cell line	Point mutation in codon 273
L3	Hela	Human cell line	Unknown
L4	Jurkat	Human cell line	Heterozygous for p53 ***
L5	SiHa	Human cell line	Unknown
L6	T24	Human cell line	Unknown
L7	SW480	Human cell line	Unknown
L8	I-37	Human lung tumor sample	Unknown
L9	I-51	Human lung tumor sample	Unknown
L10	PCS-4	Human lung tumor sample	Unknown
B1	Case 1	PBLCA	Unknown
B2	Case 2	PBLCA	Unknown
B3	Case 116	PBLCA	Unknown
B4	Case 4	PBLCA	Unknown
B5	Case 5	PBLCA	Unknown
B6	Case 152	PBLCA	Unknown
B7	Case 7	PBHS	Unknown
B8	Case 8	PBHS	Unknown

*PBLCA = Peripheral blood lung cancer affected.

**PBHS = Peripheral blood healthy subject.

***Personal communication.

**Table 3:** Detection of *TP53 *silent and missense base substitutions using DNA sequencing versus DNA microarray hybridization.

**ID**		**Genotype**	Mutation
	**DNA sequencing**	**Results of hybridization**	

P1 Pp53	248 wtCGG→mutCGC	No wild type signal: mutant probe absent	Silent
	249 wtAGG→mutAGA	No wild type signal: p-249 G_2_→T	Silent
	273 wtCGT	p-273 WT	Silent

P6 cDNA	248 wtCGG	p-248 WT	Silent
	249 wtAGG	p-249 WT	Silent
	273 wtCGT→mutCCT	No wild type signal: mutant probe absent	Proline

L1 A-427	248 wtCGG→mutCGT	p-248 WT: mutant probe absent	Silent
	249 wtAGG	p-249 WT	Silent
	273 wtCGT	p-273 WT	Silent

L2 C33 A	248 wtCGG	p-248 WT	Silent
	249 wtAGG	p-249 WT	Silent
	273 wtCGT→mutTGT	p-273 C→T	Cysteine

L4 Jurkat	248 wtCGG→mutCGK*	p-248 WT: mutant probe absent	Silent
	249 wtAGG→mutAGR**	p-249 G_2_→T: mutant probe absent p-249 WT	Silent
	273 wtCGT	p-273 WT	Silent

L7 SW480	248 wtCGG	p-248 WT	Silent
	249 wtAGG→mutAGR**	p-249 G_2_→T: mutant probe absent p-249 WT	Silent
	273 wtCGT→mutCAT	p-273 G→A	Histidine

L8 I-37	248 wtCGG→mutCGC	No wild type signal: mutant probe absent	Silent
	249 wtAGG→mutCGA	No wild type signal: mutant probe absent	Silent
	273 wtCGT→mutTGT	p-273 C→T	Cysteine

L9 I-51	248 wtCGG→mutCGC	No wild type signal: mutant probe absent	Silent
	249 wtAGG→mutCGA	No wild type signal: mutant probe absent	Silent
	273 wtCGT	p-273 WT	Silent

L10 PCS-4	248 wtCGG→mutCGC	No wild type signal: mutant probe absent	Silent
	249 wtAGG→mutCGA	No wild type signal: mutant probe absent	Silent
	273 wtCGT	p-273 WT	Silent

B3 Case 116	248 wtCGG	p-248 WT	Silent
	249 wtAGG	p-249 WT	Silent
	273 wtCGT→mutCTT	p-273 G→T	Leucine

B6 Case 152	248 wtCGG	p-248 WT	Silent
	249 wtAGG	p-249 WT	Silent
	273 wtCGT→mutCTT	p-273 G→T	Leucine

*K = G or T (Heterozygous), **R = G or A (Heterozygous)
